# Nerve Growth Factor Stimulates Interaction of Cayman Ataxia Protein BNIP-H/Caytaxin with Peptidyl-Prolyl Isomerase Pin1 in Differentiating Neurons

**DOI:** 10.1371/journal.pone.0002686

**Published:** 2008-07-16

**Authors:** Jan Paul Buschdorf, Li Li Chew, Unice Jim Kim Soh, Yih-Cherng Liou, Boon Chuan Low

**Affiliations:** Department of Biological Sciences, Faculty of Science, National University of Singapore, Singapore, Republic of Singapore; National Institutes of Health, United States of America

## Abstract

Mutations in *ATCAY* that encodes the brain-specific protein BNIP-H (or Caytaxin) lead to Cayman cerebellar ataxia. BNIP-H binds to glutaminase, a neurotransmitter-producing enzyme, and affects its activity and intracellular localization. Here we describe the identification and characterization of the binding between BNIP-H and Pin1, a peptidyl-prolyl *cis/trans* isomerase. BNIP-H interacted with Pin1 after nerve growth factor-stimulation and they co-localized in the neurites and cytosol of differentiating pheochromocytoma PC12 cells and the embryonic carcinoma P19 cells. Deletional mutagenesis revealed two cryptic binding sites within the C-terminus of BNIP-H such that single point mutants affecting the WW domain of Pin1 completely abolished their binding. Although these two sites do not contain any of the canonical Pin1-binding motifs they showed differential binding profiles to Pin1 WW domain mutants S16E, S16A and W34A, and the catalytically inert C113A of its isomerase domain. Furthermore, their direct interaction would occur only upon disrupting the ability of BNIP-H to form an intramolecular interaction by two similar regions. Furthermore, expression of Pin1 disrupted the BNIP-H/glutaminase complex formation in PC12 cells under nerve growth factor-stimulation. These results indicate that nerve growth factor may stimulate the interaction of BNIP-H with Pin1 by releasing its intramolecular inhibition. Such a mechanism could provide a post-translational regulation on the cellular activity of BNIP-H during neuronal differentiation. (213 words)

## Introduction

BNIP-H (or Caytaxin) is a brain-specific protein and mutations in its gene *ATCAY* cause human cayman cerebellar ataxia [1]. The disease is associated with hypotonia, variable psychomotor retardation, cerebellar dysfunction such as truncal ataxia and intention tremor, scoliosis, dysarthria and ocular abnormalities [2]. The same gene is also affected in three different mutant mice, *jittery, sidewinder* and *hesitant* [1], [3], [4]. *Hesitant* mice show mild ataxia and dystonia whereas *jittery* and *sidewinder* mice have severe limb and truncal ataxia, dystonic forelimb spasms and die at the age of 3–4 weeks. In rats, a mutation in *Atcay* leads to generalized dystonia [5].

We first isolated the cDNA of human BNIP-H and showed that it is required for trafficking kidney-type glutaminase (KGA) to neurites and affects the homeostasis of glutamate [6], an abundant neurotransmitter in the central nervous system, which is linked to KGA-activity [7]. BNIP-H is expressed in the spinal cord and all parts of the brain with high expression in the cerebellum and hippocampus [1], [5], [6], [8]. Therefore, deregulation of glutamate synthesis through the loss of BNIP-H function could provide an explanation for the development of cayman ataxia [6]. Xiao *et al.* [9] recently analyzed differential gene expression of dystonic and normal rats and implicated the possible involvement of phosphatidylinositol signaling pathways, calcium homeostasis and extracellular matrix interactions, while in another study, BNIP-H was found to be polyubiquitinated by the ubiquitin E3 ligase CHIP (C-terminus of Hsc70-interacting protein) *in vitro*, suggesting that BNIP-H degradation could be triggered by CHIP [10].

BNIP-H and all other BNIP-2 family proteins are characterized by their novel protein-protein interaction domain, the BNIP-2 and Cdc42GAP homology (BCH) domain [11]. In addition to their ability to confer homophilic and heterophilic interactions, the BCH domain on BNIP-2 targets the small GTPase Cdc42 [11], [12] leading to cell elongation and protrusion [13] whereas BNIP-Sα induces cell rounding and apoptosis by engaging RhoA and displacing its inactivator, p50RhoGAP [14], [15]. On the other hand, BPGAP1 utilizes its BCH domain to promote protrusion and cell migration [16], [17] as well as Ras-MAPK signaling [18], pointing to the versatility of BCH-domain in regulating diverse cellular processes. However, it remains unclear how the activity of BCH domains is controlled in distinct cellular conditions.

We recently found that certain regions of the BNIP-H BCH domain could form an intramolecular interaction, implicating that it might be subjected to protein folding or conformational control. Furthermore, its N-terminus harbors several putative serine/threonine residues followed by proline, a possible target for the group IV WW-domains [19]. To this end, we have performed a “candidate” approach screening and identified Pin1, a WW domain-containing peptidyl-prolyl cis/trans-isomerase as a putative binding partner of BNIP-H. Pin1 contains an N-terminal WW domain, which recognizes and binds a core motif consisting of a phosphorylated serine or threonine followed by a proline residue [20] while its C-terminal peptidyl-prolyl cis/trans-isomerase (PPI) domain catalyzes the cis/trans-isomerization of the peptide bond in the center of the core binding motif [21]. Thereby, Pin1 impacts the structure, catalytic activity, phosphorylation status and stability of many proteins involved in various cellular processes [22], [23], including cell cycle control [24], [25], transcription [26], [27] and apoptosis [28]–[30]. Furthermore, Pin1 was also shown to be involved in neurodegenerative disorders such as the Alzheimer's disease [31], [32], Parkinson disease [33], amyotrophic lateral sclerosis [34], and in cancer [35].

We have characterized the interaction between BNIP-H and Pin1 in differentiating neurons and have further shown that NGF-stimulation of PC12 cells strongly enhanced their interaction via distinct binding motifs, including two atypical sites for WW domain-binding and the catalytic PPI domain but with differential binding profiles. This interaction is facilitated by the release of the intramolecular binding within BNIP-H, supporting the notion that NGF could stimulate their interaction by removing such inhibition. Furthermore, Pin1 disrupted the BNIP-H/glutaminase-binding in PC12 cells under nerve growth factor-stimulation. Our results therefore reveal a possible post-translational regulation of BNIP-H by Pin1 during neuronal differentiation.

## Results

### BNIP-H binds to Pin1 after NGF-stimulation in PC12 cells

**Figure 1:**
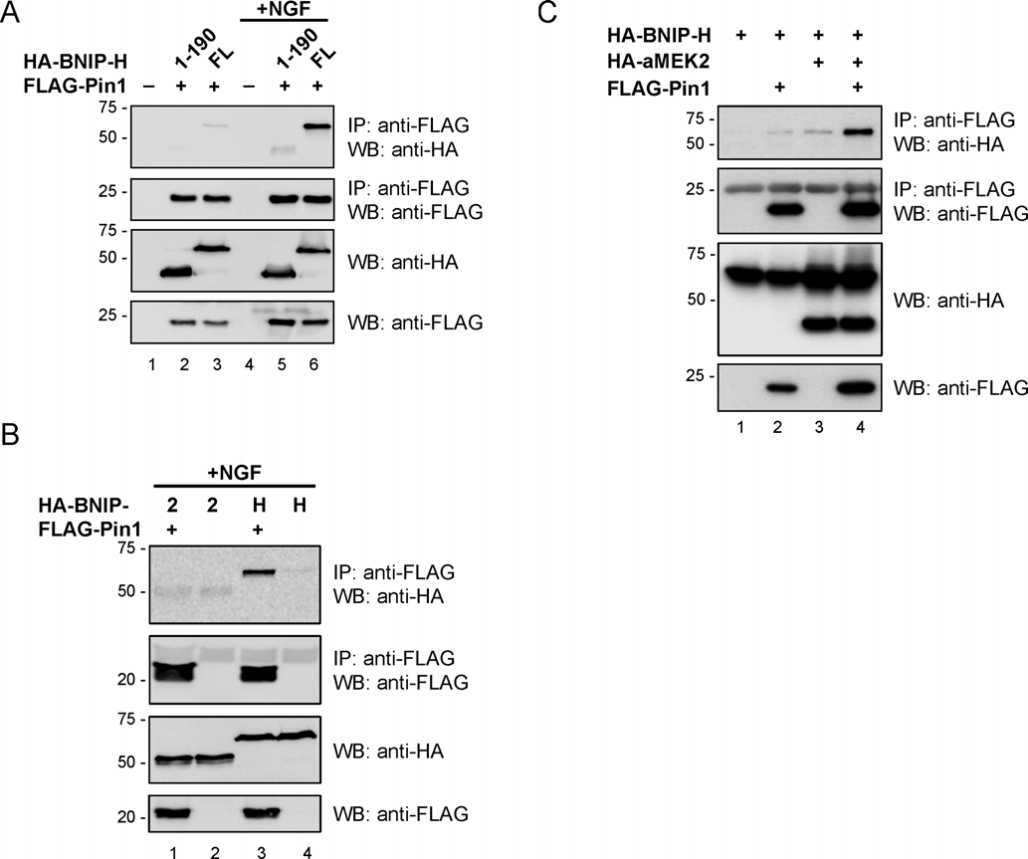
BNIP-H, but not BNIP-2 binds Pin1 after NGF-stimulation in PC12 cells. (A) PC12 cells were transfected with expression plasmids for HA-BNIP-H full length or HA-BNIP-H aa 1–190 and FLAG-Pin1 and either treated with NGF or left unstimulated. After immunoprecipitation with anti-FLAG antibody, samples were analyzed by western-blotting with the indicated antibodies to reveal binding of BNIP-H. (B) PC12 cells were transfected with expression plasmids for HA-BNIP-2 or HA-BNIP-H alone or together with an expression construct for FLAG-Pin1 and treated with NGF. After immunoprecipitation with anti-FLAG antibody, samples were analyzed by western-blotting with the indicated antibodies to reveal binding of BNIP-2 or BNIP-H. (C) 293T cells were transfected with an expression construct for HA-BNIP-H with or without FLAG-Pin1 expression plasmids (lanes 1 and 2), and for HA-BNIP-H and the constitutively activated form of HA-MEK2 with or without FLAG-Pin1 plasmid (lanes 3 and 4). After immunoprecipitation with anti-FLAG antibody, precipitates and whole cell lysates were analyzed by western-blotting with the indicated antibodies to reveal binding of HA-BNIP-H.

BNIP-H contains a C-terminal BCH domain (aa 190–332) and an N-terminus (aa 1–189) with no obvious similarity to any known protein domain. However, its N-terminus contains several serine and threonine residues followed by proline, which are potential binding motifs for Pin1 when phosphorylated [20]. The pheochromocytoma cell line PC12 responds to NGF-treatment with the development of a neuron-like phenotype and phosphorylation of various proteins [36]. To examine their possible interaction in differentiating PC12 cells, HA-BNIP-H full length or the N-terminal BNIP-H fragment (aa 1–190) were co-expressed with FLAG-Pin1 in PC12 cells, in the presence or absence of the NGF. The result shows that BNIP-H full length co-immunoprecipitated strongly with Pin1 only after NGF treatment. In the absence of NGF very weak binding was detected while the N-terminal BNIP-H fragment did not show any interaction in either condition (Fig. 1A). These data suggest that the binding between BNIP-H and Pin1 in PC12 cells is induced by NGF. Unexpectedly, the result also revealed that its C-terminus that contains the BCH domain but not the N-terminus (with its potential binding motifs) is crucial for this interaction. To test the specificity of such interaction, BNIP-2, a closely related protein that shares 52% amino acid sequence identity (69% amino acid similarity) with BNIP-H, was tested to examine if it would also bind to Pin1. PC12 cells were transfected with expression plasmids for HA-BNIP-2 or HA-BNIP-H alone or together with a construct for FLAG-Pin1, treated with NGF and subjected to immunoprecipitation. BNIP-H, but not BNIP-2, co-immunoprecipitated with Pin1 (Fig. 1B), suggesting a specific interaction despite the close homology of these two proteins.

To confirm that the binding between the two proteins is indeed dependent on stimulation, we assessed the formation of the BNIP-H/Pin1 complex in the presence and absence of constitutively activated MEK2. It is activated through NGF-stimulation in PC12 cells and therefore a constitutively activated MEK2 might be able to mimic stimulation that leads to the complex formation in 293T cells. To this end, we expressed HA-BNIP-H full length and the constitutively activated form of MEK2 with or without FLAG-Pin1 in 293T cells. In another set, we expressed HA-BNIP-H with or without FLAG-Pin1 in the absence of constitutively activated MEK2. After immunoprecipitation with anti-FLAG antibody and western-blot analysis of precipitates and cell lysates, a strong signal for HA-BNIP-H was detected in the precipitate of cells that had been transfected with expression constructs for BNIP-H, Pin1 and constitutively activated MEK2 (Fig. 1A, lane 4). Only a faint signal was detected in the corresponding sample without constitutively activated MEK2 (lane 2). These results confirm that the binding between BNIP-H and Pin1 is dependent on stimulation. In the case of PC12 cells, this stimulation can be triggered by NGF (Fig. 1A).

### BNIP-H and Pin1 co-localize in differentiating neuron-like cells

**Figure 2:**
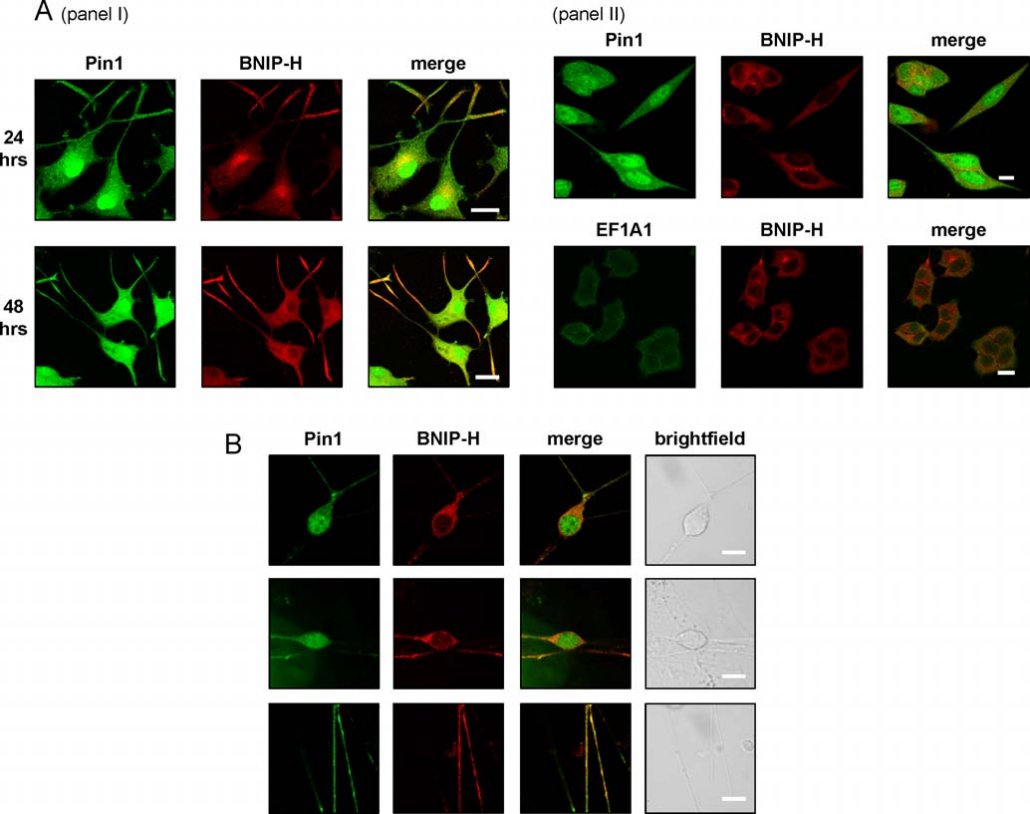
BNIP-H co-localizes with Pin1 in differentiating PC12 and P19 cells. PC12 (A) and P19 cells (B) were fixed, permeabilized and probed with the indicated antibodies, followed by appropriate fluorophore-conjugated secondary antibodies and analyzed by confocal microscopy. PC12 cells were treated with NGF for 24 and 48 hours, respectively (panel I). Undifferentiated PC12 cells grown for 48 hours were used in panel II. P19 cells were treated with retinoic acid for five days and allowed to differentiate for another six days without retinoic acid. Scale bar in (A) 20 µm for panel I, and 10 µm for panel II, in (B) 10 µm.

Next, we set out to examine the localization of endogenous Pin1 and BNIP-H by using confocal microscopy and immunofluorescence analysis on PC12 and P19 cells. Firstly, PC12 cells were treated with NGF for 24 or 48 hours, fixed and then stained for endogenous BNIP-H and Pin1 (Fig. 2A, panel I). After 24 hours, BNIP-H was localized at the perinuclear region and the endings of neurites. Pin1 was predominantly found in the nucleus but was also localized at the perinuclear region and in neurites where it co-localized with BNIP-H. After 24 hours, BNIP-H was localized at the perinuclear region and the endings of neurites. Pin1 was mainly localized to the nucleus but was also found perinuclear and in neurites where it co-localized with BNIP-H. After 48 hours of NGF treatment, BNIP-H and Pin1 showed an almost even cytoplasmic distribution. Importantly, both proteins mostly co-localized in the cytoplasm, neurites and neurite terminals, except that some pools of Pin1 still resided in the nucleus without any sign of BNIP-H there. This result indicates that only selective pools of Pin1 were targeted by BNIP-H. As a control for random co-localization, we used PC12 cells grown in the absence of NGF for 48 hours, and stained for endogenous BNIP-H and Pin1, or for endogenous BNIP-H and an unrelated protein, elongation factor 1A1 (EF1A1). Only minimal co-localization was observed for BNIP-H and Pin1 without NGF (Fig. 2A, panel II). This result is in agreement with data presented in Fig. 1A, which shows that the binding between BNIP-H and Pin1 is strongly enhanced upon NGF-stimulation. As a further control, we detected endogenous BNIP-H and EF1A1 and found no co-localization between the two proteins (Fig. 2A, panel II). The embryonic carcinoma cell line P19 was also examined for the localization of BNIP-H and Pin1. P19 cells were treated with retinoic acid for five days to induce neuronal differentiation. After another five days of differentiation, the cells were fixed and stained for BNIP-H and Pin1 (Fig. 2B). BNIP-H was localized to the cytoplasm and neurites. Again, Pin1 was mainly localized to the nucleus but was also found in the cytoplasm and in neurites where it co-localized with BNIP-H. Taken together, these results suggest that BNIP-H interacts with Pin1 during neuronal differentiation.

### BNIP-H binds to the WW domain of Pin1

**Figure 3:**
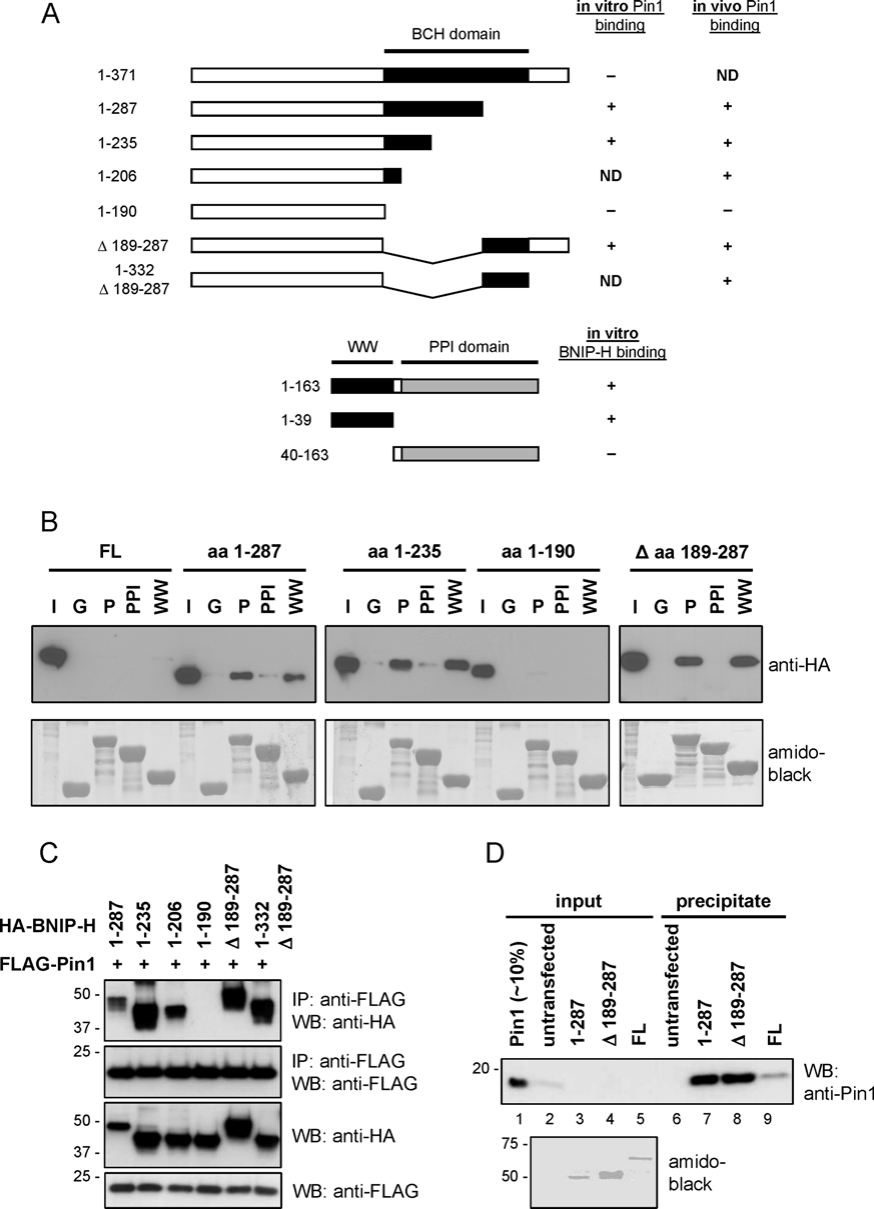
BNIP-H contains two Pin1-binding sites and interacts with the WW domain of Pin1. Purified GST fusion proteins of Pin1 full length, Pin1 WW domain and Pin1 PPI domain were incubated with PC12 whole cell lysates containing either HA-BNIP-H full length or various BNIP-H deletions proteins, which were expressed under NGF-stimulation (A, *in vitro* binding). After incubation, GST fusion proteins were isolated, washed and analyzed for the presence of bound BNIP-H or its mutants by western-blotting with anti-HA antibody. The membrane with the blotted proteins was than stained with amido black to reveal equal loading of GST and GST fusion proteins. GST protein was used as a control. Input shows approximately 2% of the lysate used for the GST pull-down assay. I, input; G, GST; P, Pin1 full length; WW, WW domain of Pin1; PPI, PPI domain of Pin1 (B). Various HA-tagged BNIP-H expression constructs (A, *in vivo* binding) and a FLAG-Pin1 expression plasmid were co-transfected into 293T cells. Lysates were subjected to immunoprecipitation with anti-FLAG antibody. Precipitates and whole cell lysates were analyzed by western-blotting with the indicated antibodies to detect binding of the different HA-BNIP-H fragments. A summary of the results is presented (A, *in vivo* binding) (C). 293T cells were transfected with expression plasmids for FLAG-BNIP-H full length, FLAG-BNIP-H aa 1–287, FLAG-BNIP-H Δ aa 189–287 or left untransfected. Lysates were subjected to immunoprecipitation with anti-FLAG antibody conjugated to agarose beads, and precipitates were washed thoroughly with RIPA buffer and RIPA buffer supplemented with 0.1–0.5% sodium dodecyl sulfate or 500 mM NaCl as described under “Material and Methods”. Precipitates were resuspended in lysis buffer and incubated with recombinant Pin1 expressed and purified from *E.coli*. After sedimentation and washing, precipitates were analyzed by western-blotting with anti-Pin1 antibody (lanes 6–9). Lane 1 shows 10% of the input for recombinant Pin1. Lanes 2–5 demonstrate that the purified FLAG-BNIP-H constructs were devoid of endogenous Pin1 from 293T cells. The amido black-stained membrane shows equal input for the FLAG-BNIP-H constructs.

In order to understand the mechanism underlying this interaction, the various binding regions within Pin1 and BNIP-H were first determined by GST pull-down assays. GST fusion proteins of Pin1 full length, Pin1 WW domain and Pin1 PPI domain were expressed in *E.coli*, purified and then incubated with lysates prepared from PC12 cells expressing either HA-BNIP-H full length or various BNIP-H deletions proteins, all under NGF-stimulation (Fig. 3A, *in vitro* binding). After incubation, the GST fusion proteins were isolated, washed and analyzed for the presence of bound BNIP-H or its mutants. Interestingly, no signals were detected for BNIP-H full length incubated with GST-Pin1 full length, GST-Pin1 WW domain or GST-Pin1 PPI domain (Fig. 3B). Only after prolonged exposure, weak binding towards Pin1 full length and Pin1 WW domain was observed (data not shown). Interestingly, C-terminal deletion of BNIP-H (fragments aa 1–287 and aa 1–235) exhibited strong interaction with Pin1 full length and Pin1 WW domain but negligible interaction with the PPI domain. This apparent lack of interaction with the PPI domain turned out to be a transient one and could only be captured by the catalytically inert version of PPI (see below). However, further C-terminal deletion of the BCH domain (fragment aa 1–190) resulted in the complete loss of binding. In comparison, a BNIP-H mutant with an internal deletion of aa 189–287 still showed strong binding towards Pin1 full length and Pin1 WW domain. Taken together, these results suggest that there are at least two Pin1-binding sites within BNIP-H: one that is located between aa 190 and 235 in the BCH domain (binding site 1) while another binding site is in the C-terminus of BNIP-H between aa 287 and 371 (binding site 2). Interestingly, neither of these regions contained contain a serine/threonine-proline motif, which could have served as potential canonical binding site for Pin1. Further, the key binding domains of Pin1 for BNIP-H lie at the WW domain (and PPI also, see next section). A schematic summary of all the results obtained from the GST pull-down assays is shown in Fig. 3A (*in vitro* binding). The absence of strong binding of BNIP-H full length to Pin1 is possibly due to the *in vitro* condition of the GST pull-down assay. To further define the two Pin1-binding sites in BNIP-H, we employed co-immunoprecipitation studies with FLAG-Pin1 full length and, in addition to the set of HA-tagged BNIP-H constructs that were used in the GST pull-down assay (except HA-BNIP-H full length), two more constructs: HA-BNIP-H aa 1–206 and HA-BNIP-H aa 1–332 Δ189–287 (Fig. 3A, *in vivo* binding). 293T cells were co-transfected with expression plasmids for FLAG-Pin1 full length and different HA-BNIP-H constructs. After immunoprecipitation and western-blot analysis, it was found that all BNIP-H constructs except aa 1–190, were able to bind to Pin1 (Fig. 3C). The results suggest that binding site 1 is located between aa 191–206, and binding site 2 lies between aa 287 and 332. Weak or no signals were observed when the HA-BNIP-H constructs were subjected to immunoprecipitation in the absence of FLAG-Pin1 (data not shown). A summary of the results is presented in Fig. 3A (*in vivo* binding). These data are in agreement with the results of the GST pull-down assays, and further narrow down the two Pin1-binding sites to a region of 16 aa (binding site 1) and 45 aa (binding site 2), respectively.

Next, to determine whether the BNIP-H/Pin1 interaction was primarily mediated by direct binding and not via an intermediary protein, different BNIP-H truncations were expressed in *E.coli* or an in vitro transcription and translation system, however we failed to detect expression in either system (data not shown). Instead, the FLAG-BNIP-H full length, FLAG-BNIP-H aa 1–287 and FLAG-BNIP-H Δ aa 189–287 were expressed in 293T cells and purified by immunoprecipitation and subsequent washing in buffers containing 0.1–0.5% SDS or 650 mM NaCl as outlined in “Material and Methods”. In addition to FLAG-BNIP-H full length, we included untransfected 293T cells as another control. All samples were incubated with recombinant Pin1 that had been expressed and purified from *E.coli*. After precipitation of the FLAG-tagged constructs and thorough washing, all samples were analyzed by western-blotting with an anti-Pin1 antibody. Fig. 3D shows that Pin1 was strongly bound by FLAG-BNIP-H aa 1–287 and FLAG-BNIP-H Δ aa 189–287 (lanes 7 and 8), but only a weak or no signal was detected with FLAG-BNIP-H full length and untransfected cells respectively (lanes 6 and 9). As the FLAG-BNIP-H constructs were purified from a human cell line, we therefore included lanes 2–5 in Fig. 3D to demonstrate that all constructs were devoid of endogenous Pin1 after purification. The results demonstrate that BNIP-H binds directly to Pin1 *in vitro*. Furthermore, this assay also confirms that without stimulation both proteins do not form a complex, thus in agreement with data presented in Fig. 1A and 1C.

### Intramolecular interaction within BNIP-H

**Figure 4:**
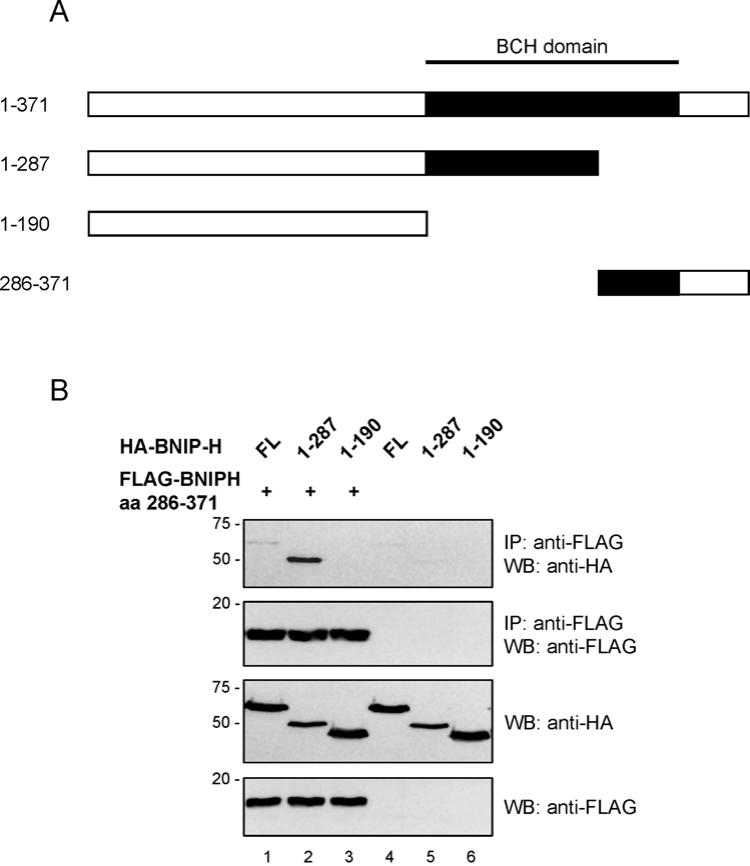
Intramolecular binding in BNIP-H. 293T cells were transfected with expression plasmids for HA-BNIP-H full length, HA-BNIP-H aa 1–287 or HA-BNIP-H aa 1–190 alone or together with an expression construct for a FLAG-tagged C-terminal BNIP-H fragment (aa 286–371). A schematic picture of these fragments is shown (A). After co-immunoprecipitation with anti-FLAG antibody samples were analyzed by western-blotting with the indicated antibodies to reveal binding of the different HA-tagged fragments (B).

The ability of the C-terminal deletion or internal deletion of BNIP-H, but not the full length BNIP-H to interact with Pin1, strongly suggests that the binding sites within BNIP-H full length are not readily accessible. This is probably due to some steric hindrance as a result of an intramolecular interaction. C-terminal deletion and internal deletion would have exposed such binding sites. Consequently, NGF-stimulation might relieve such intramolecular binding while promoting Pin1-binding within the cells. To further characterize this intramolecular interaction in intact cells, FLAG-tagged C-terminal BNIP-H fragment (aa 286–371) was co-expressed with either HA-BNIP-H full length, HA-BNIP-H aa 1–287 or HA-BNIP-H aa 1–190 in 293T cells and their respective lysates subjected to co-immunoprecipitation. A schematic picture of the BNIP-H fragments used is shown in Fig. 4A. The C-terminal BNIP-H fragment (aa 286–371) did not bind to HA-BNIP-H aa 1–190 but showed strong binding towards aa 1–287 of BNIP-H (Fig. 4B). Interestingly, only faint signals were observed for binding towards the full length protein. These data strongly show that the intramolecular interaction occurs between the proximal and distal part of the BCH domain. The absence of binding to BNIP-H full length suggests that the full length protein is likely to be expressed in a closed conformation and that the fragment aa 286–371 cannot compete off the intramolecular binding of the same region. Interestingly, the two regions of BNIP-H, which bound to each other via this intramolecular interaction, also harbor the two binding sites for Pin1. The specific motifs responsible for these two distinctive bindings are not yet known and await further investigation. All these results therefore suggest that NGF-stimulation could cause the release of intramolecular inhibition on BNIP-H, thus leading to the exposure of the actual Pin1-binding sites to facilitate the interaction between BNIP-H and Pin1.

### The two Pin1-binding sites on BNIP-H have distinctive binding profiles

The results presented in Fig. 3C demonstrate that if the Pin1-binding sites become more readily accessible through C-terminal deletion or internal deletion, Pin1 inside the cells binds to the truncated BNIP-H fragment aa 1–287 and the internally deleted fragment Δ aa 189–287 without any stimulation. In agreement with this, we showed that both regions bound to each other when expressed separately in 293T cells (Fig. 4B) and that almost no binding was observed between BNIP-H full length and Pin1 in 293T cells in the absence of a stimulus (Fig. 1C). Thus, the two BNIP-H fragments, aa 1–287 and the internally deleted fragment Δ aa 189–287 can be used to individually characterize the two Pin1-binding sites in the absence of stimulation.

**Figure 5:**
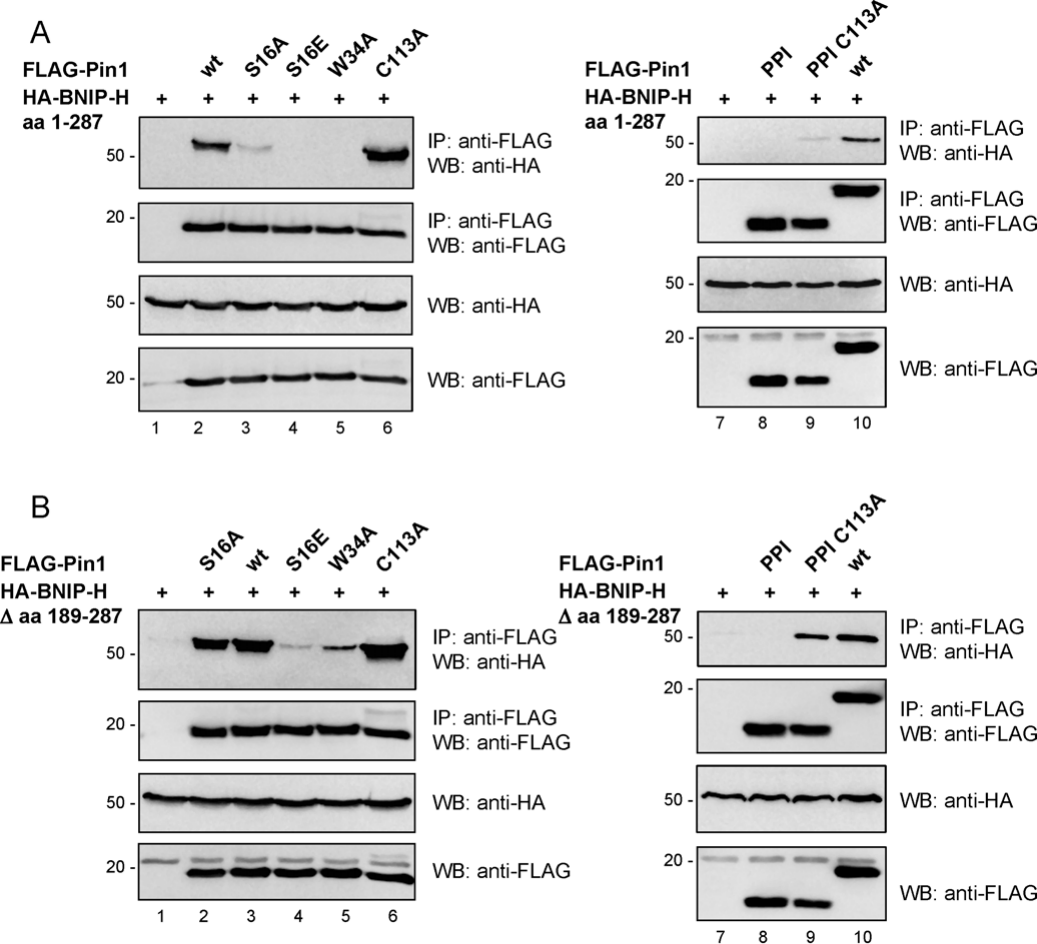
The two Pin1-binding sites within BNIP-H have different binding profiles. 293T cells were transfected with expression plasmids for HA-BNIP-H aa 1–287 (A) or HA-BNIP-H Δ aa 189–287 (B) alone or together with expression plasmids for full length FLAG-tagged Pin1 wt, different full length FLAG-tagged point mutants or expression plasmids for FLAG-tagged PPI domain of Pin1 with or without the point mutation C113A. After immunoprecipitation with anti-FLAG antibody, samples were analyzed by western-blotting with the indicated antibodies to reveal binding of the two different HA-tagged BNIP-H fragments.

The presence of two binding regions for seemingly only one WW domain of Pin1 as shown by *in vitro* pull-down assays and co-immunoprecipitation raises the question on (i) the relative contribution by these two regions when binding to Pin1, and (ii) the role of PPI domain of Pin1 in the formation of Pin1-BNIP-H complex *in vivo*. To address these two issues, different Pin1 mutants affecting the WW domain (S16A, S16E and W34A) [37], [38] or the PPI domain (C113A) [37] were employed to determine the binding profile for the two binding sites on BNIP-H. 293T cells expressing HA-BNIP-H aa 1–287 or HA-BNIP-H Δ aa 189–287 in the presence of different FLAG-Pin1 mutants were subjected to co-immunoprecipitation followed by western-blot analysis. As shown in Fig. 5A, BNIP-H aa 1–287 only bound to the wild-type Pin1 and not to any of the Pin1 mutants affecting the WW domain. The binding was moderately increased by the Pin1 mutant C113A (Fig. 5A, lanes 3–6), indicating the absolute requirement for the WW domain of Pin1. In contrast, the Pin1 S16A mutant and to a lesser extent the W34A mutant, still retained binding towards BNIP-H Δ aa 189–287. Similar to BNIP-H aa 1–287, no significant binding of BNIP-H Δ aa 189–287 towards the Pin1 S16E mutant was detected. Again, a strong interaction was observed with the Pin1 mutant C113A (Fig. 5B, lanes 2 and 4–6). These results demonstrate that the two Pin1-binding sites within BNIP-H have common as well as different binding properties towards Pin1.

We next tested the binding of the two BNIP-H fragments towards the PPI domain with or without the C113A mutation. The BNIP-H fragment aa 1–287 did not bind to the PPI domain with or without the C113A mutation (Fig. 5A, lanes 8 and 9). The BNIP-H fragment Δ aa 189–287 did not bind to the PPI domain either, but it showed binding towards the PPI domain carrying the C113A mutation (Fig. 5B, lanes 8 and 9). For each fragment a positive control was included, which was the binding to Pin1 full length (Fig. 5A and B, lane 10). The absence of binding between the two BNIP-H fragments and the wild-type PPI domain implies that the first step in the interaction of Pin1 with BNIP-H is likely to be mediated by the WW domain. In a second step the binding could be mediated by the PPI domain as indirectly shown by enhanced binding to either the mutant PPI domain (as for the BNIP-H fragment Δ aa 189–287) or full length Pin1 carrying the C113A mutation (as for the BNIP-H fragment aa 1–287; Fig. 5A, compare lanes 2 and 6).

Taken together, our results reveal an unexpected interaction between BNIP-H and Pin1, requiring at least two distinctive regions in the C-terminus of BNIP-H that show differential binding profiles towards the full-length and PPI domain of Pin1 (summarized in Table 1).

**Table 1:** Binding profile of Pin1-binding sites 1 and 2 in BNIP-H.

		BNIP-H aa 1–287	BNIP-H Δ aa189–287
Pin1 full length	WT	+	+
	S16A	–	+
	S16E	–	–
	W34A	–	(+)
	C113A	++	++
PPI domain	WT	–	–
	C113A	–	+

The binding between different forms of BNIP-H and Pin1 are as detailed in the text. The + sign denotes relative strength of interactions as determined by co-immunoprecipitation studies. BNIP-H aa 1–287 harbors binding site 1 (aa 191–206) and BNIP-H Δ aa 189–287 contains binding site 2 (aa 287–332).

### Pin1 competes with glutaminase for binding to BNIP-H

**Figure 6:**
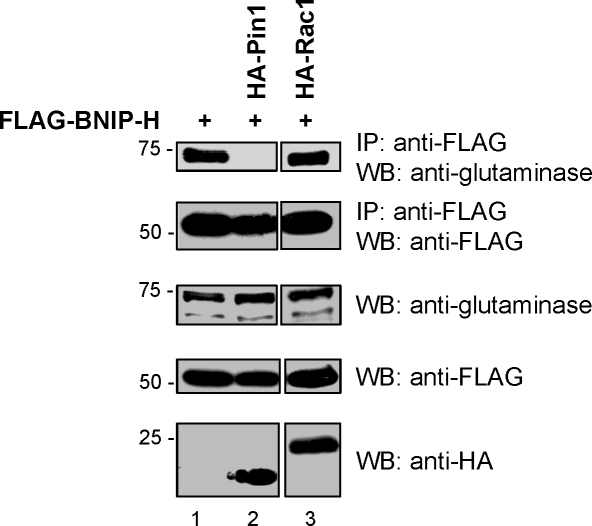
Pin1 competes with glutaminase for binding to BNIP-H in PC12 cells. PC12 cells were transfected with an expression plasmid for FLAG-BNIP-H full length alone or together with constructs for HA-Pin1 or HA-Rac1 as indicated. Cells were treated with NGF for 24 hours. After immunoprecipitation with anti-FLAG antibody, samples were analyzed by western-blotting with the indicated antibodies to reveal binding of endogenous kidney-type glutaminase to BNIP-H in the absence (lane 1) or presence of Pin1 (lane 2) or Rac1(lane 3). Samples shown were from the same experiment and analyzed on a single blot.

We have previously shown that BNIP-H binds kidney-type glutaminase and regulates its enzyme activity [6], which is essential for the production of the neurotransmitter glutamate [7]. Kidney-type glutaminase binds to two regions within BNIP-H, which essentially overlap with binding sites 1 and 2 for Pin1 [6]. We therefore hypothesized that Pin1 could compete with glutaminase for binding to BNIP-H. We expressed FLAG-BNIP-H and HA-Pin1 in PC12 cells in the presence of NGF. A HA-tagged small GTPase Rac1, which does not bind to BNIP-H was used as a negative control. After immunoprecipitation with anti-FLAG antibody precipitates were examined for the presence of endogenous kidney-type glutaminase. Glutaminase was co-precipitated when FLAG-BNIP-H was expressed alone (Fig. 6, lane 1) or together with HA-Rac1 (lane 3) in PC12 cells under NGF-stimulation. However, in the presence of exogenously expressed HA-Pin1 no glutaminase was detected in the precipitate (lane2). These data are in agreement with the overlapping binding sites for glutaminase and Pin1 and support the role of Pin1 in regulating the binding of glutaminase to BNIP-H in PC12 cells in the presence of NGF. The significance of this is discussed.

## Discussion

Cayman cerebellar ataxia is caused by mutations in the gene for BNIP-H, which contains a novel protein binding domain, the BCH domain [12]. To date, only two binding partners for BNIP-H have been identified: the ubiquitin E3 ligase CHIP [10] and the neurotransmitter-producing enzyme, the kidney-type glutaminase [6]. The activities of both enzymes need to be tightly regulated for proper cell function. In the case of glutaminase, we have shown that BNIP-H is able to inhibit its enzyme activity as well as to relocalize glutaminase from mitochondria in the cell body to neurites [6]. The question arises as to how BNIP-H is in turn regulated to execute its control on the activity and relocalization of glutaminase that is needed in neurons. One obvious mechanism is protein degradation triggered by ubiquitination as suggested by the identification of CHIP as a putative BNIP-H binding partner [10]. Here, we present evidence for an interaction between BNIP-H and Pin1 that is induced by NGF-stimulation in PC12 cells. The binding occurs via two cryptic regions within BNIP-H's C-terminus encompassing the BCH domain. In addition, we showed intramolecular binding within the C-terminus of BNIP-H, which includes the two regions important for Pin1-binding. Our results suggest that NGF-stimulation could cause the relief of intramolecular inhibition/binding within BNIP-H. The subsequent exposure of the two Pin1-binding sites will then facilitate interactions with Pin1 mediated by its WW domain and then via its PPI domain in differentiating neurons. Conformational changes induced by NGF-stimulation and/or possibly the PPI domain of Pin1 could provide a mechanism of post-translational modification of BNIP-H. It remains to be seen how such interaction could alter BNIP-H's affinity for its other binding partners such as the glutaminase, thus indirectly influencing the production of the neurotransmitter glutamate as well as its spatial distribution. Our immunofluorescence studies indicate that BNIP-H follows the distribution of Pin1 outside the nucleus suggesting that Pin1 could be an upstream regulator of BNIP-H. Similarly, in the case of CHIP, conformational changes could modify the rate of BNIP-H degradation triggered by ubiquitination. While both mechanisms could be important modes for regulating BNIP-H's activity during brain development, the actual effects of Pin1-binding to BNIP-H on regulating glutaminase activity and CHIP-linked protein degradation await further investigation.

We have identified two binding regions for Pin1 in the C-terminus of BNIP-H. These regions overlap with the two sites previously identified for glutaminase-binding to BNIP-H [6]. An important difference between the binding profiles of BNIP-H with these two proteins is that glutaminase-binding does not seem to be dependent on NGF-stimulation [6]. It remains an interesting question as to whether Pin1 and glutaminase are functionally connected through BNIP-H or if they bind to different pools and act independently from each other. Our results presented in Fig. 6 suggest that Pin1 is able to compete off the binding of glutaminase to BNIP-H. This competition would only occur under growth factor-stimulation and could present a mechanism to regulate the release of glutaminase from BNIP-H. Alternatively, Pin1 might affect the post-translational modification of BNIP-H (and/or glutaminase) resulting in the loss of interaction between BNIP-H and glutaminase. Since BNIP-H regulates the activity and localization of kidney-type glutaminase [6], such mechanisms could indirectly control its distribution and/or glutamate synthesis. The presence of two Pin1-binding sites with a different binding profile also raises the key issue as to which site contributes more to the interaction with Pin1 and their biological significance. It could be possible that the WW domain of Pin1 binds to one site whereas the PPI domain targets the other. This model is in contrast to proteins with only one common binding site where WW domain and PPI domain are believed to act on the same motif [23]. The WW domain is a well characterized protein binding domain, and for Pin1, it is important for its nuclear localization [39], [40]. The core binding motif for Pin1 is a phosphorylated serine or threonine residue followed by proline [21], [41]. Interestingly, both binding regions in human BNIP-H (aa 191–206 and aa 286–332) do not contain such a motif, suggesting a non-canonical binding between BNIP-H and Pin1. The S16E Pin1 mutant did not bind to both binding sites in the co-immunoprecipitation studies, indicating that the interaction could be disrupted by the presence of negative charge. In contrast, the binding of the S16A Pin1 mutant to binding site 2 but not binding site 1, suggests that the hydroxyl group of the serine residue is important for the interaction at binding site 1 but dispensable for the other. In relation to this, the WW domain of Pin1 harboring the S16A mutation is still able to bind to phosphoproteins [20] whereas its S16E mutation does not bind p53 suggesting its inability to bind phosphoproteins [42]. The basis for the selectivity and the significance of the serine-16 of Pin1 in binding to BNIP-H awaits further characterization. In comparison, the PPI mutant C113A was shown to have low PPI activity [37], most probably due to the involvement of the C113 residue in the catalytic mechanism of prolyl-isomerisation by forming covalent interactions with the substrate as suggested by Ranganathan et al. [41]. We speculate that since the binding between BNIP-H and Pin1 could be transient (kiss-and-run), it could slow down the release of the product after being recognized, thus leading to enhanced binding to BNIP-H as shown in Fig. 5A and B. This would imply that BNIP-H is a substrate of Pin1; however detailed enzymatic studies are needed to clarify this point.

One of the most critical questions that needs to be addressed is the identification of the exact binding sequence of both binding sites within BNIP-H. We have narrowed down the region of binding site 1 to 16 amino acids whereas binding site 2 is located in a region of 45 amino acids, but neither of which have the typical Pin1-binding motifs. Indeed, recent publications demonstrate that Pin1 is able to bind to non-canonical motifs such as pThr-Gly in cyclin E [43] and that Pin1 isomerizes cyclin E at a non-canonical proline-proline bond located in close proximity to its binding site [44]. However, such sequence motifs are also absent from these two putative sites, suggesting that other potential non-canonical motifs may exist on BNIP-H for Pin1-recognition. Li et al. reported an interaction between the protein phosphatase inhibitor Inhibitor-2 and Pin1 that is governed by the phosphorylation status of both proteins [45]. Their results show that phosphorylation of the recognition motif Pro-X-Thr-Pro of Inhibitor-2 is not required for binding to Pin1. In addition, their data suggest that Inhibitor-2 alters the binding specificity of Pin1 to phosphoproteins. Further investigation should identify the binding motifs for both sites to fully characterize the non-canonical binding between BNIP-H and Pin1, and should allow further dissection of their actual biological consequence at the cellular and developmental level.

Our findings also revealed a previously unknown regulation of Pin1-binding to its partners under NGF-regime, raising the possibility that the function of Pin1 can be further regulated by growth factor-signaling, especially in neuronal development. More specifically, enhanced BNIP-H binding to Pin1 under such conditions strongly suggests that their function could be coupled to the maturation of neurons. While the signaling mechanism underlying this stimulation of interaction needs to be explored, our results highlight the plasticity of the BCH domain in conferring post-translational regulation of BNIP-H activity in the cells.

In summary, we have identified BNIP-H/Pin1 as a novel NGF-inducible biological complex and further delineated the presence of multiple binding regions that do not bear the canonical motif for either the WW or PPI domain of Pin1. As both proteins are key determinants for brain development and neuronal function, this interaction could exert an important post-translational checkpoint on the activity of BNIP-H and its other protein associates.

## Materials and Methods

### Plasmids

The FLAG- and HA-pXJ40 expression vectors were from Ed Manser (Institute for Molecular and Cell Biology, Singapore). Human BNIP-H and Pin1 expression constructs were generated through polymerase chain reaction (PCR) with appropriate primers containing either a BamHI or XhoI restriction site for cloning into the FLAG- and HA-pXJ40 expression vectors. cDNAs encoding for Pin1 full length, Pin1 WW domain and Pin1 PPI domain, respectively, were generated through PCR and cloned into pGEX 4T1 (Amersham Biosciences) for bacterial expression of GST fusion proteins. Constructs were verified through sequencing and propagated in *E.coli* strains XL1-Blue and DH5α. For experiments shown in Fig. 1 a triple FLAG-tagged Pin1 expression construct was used (Sigma). The expression construct for constitutively activated MEK2 (HA-tagged) was purchased from Upstate Biotechnology.

### Cell culture and transfection

Human 293T cells were cultured in RPMI 1640 supplemented with 10% fetal bovine serum (Hyclone). Cells at 60–80% confluence in six-well plates were transfected with 1–2 µg plasmid DNA using Fugene 6 cationic lipid (Roche), according to the manufacturer's instructions. PC12 cells were cultured in DMEM supplemented with 5% fetal bovine serum (Hyclone) and 10% horse serum (Gibco). PC12 cells on poly-D-lysine-coated surfaces in six well plates were transfected with Lipofectamine 2000 Reagent (Invitrogen), according to the manufacturer's instructions. Differentiation was induced with 40 ng/ml nerve growth factor in the presence of 0.5% serum. P19 cells were cultured in alpha-modified Minimal Essential Medium supplemented with 7.5% bovine serum (both from Gibco) and 2.5% fetal bovine serum (Hyclone) and differentiated as described previously [6], [46].

### GST pull-down assay

Expression of GST and GST fusion proteins of Pin1 full length, Pin1 WW domain and Pin1 PPI domain in *E.coli* strain XL1-Blue was induced with 0.1 mM IPTG at room temperature overnight. Cells were harvested by centrifugation, resuspended in lysis buffer (PBS with 1% Triton X-100, 0.1 mM DTT and a mixture of protease inhibitors) and lysed by sonication. After centrifugation, the supernatant was incubated with Glutathione-Sepharose beads (Amersham Biosciences) at 4°C overnight to capture the GST fusion proteins. Beads were washed five times with lysis buffer and resupended in PBS plus protease inhibitors. HA-tagged BNIP-H or various deletion mutants were expressed in nerve growth factor-treated PC12 cells. Cells were lysed with RIPA buffer: 50 mM Tris-HCl pH 7.3, 150 mM NaCl, 0.75 mM EDTA, 1% sodium deoxycholate, 1% Triton-X-100, 0.2% sodium fluoride, 25 mM glycerol 2-phosphate, 5 mM sodium orthovanadate and a mixture of protease inhibitors (Roche). After centrifugation, the lysate was incubated with the Glutathione-Sepharose beads coated with 5 µg of GST alone or different GST-Pin1 fusion proteins for 6 hours at 4°C. The beads were washed three times with RIPA buffer and analyzed by western-blotting with anti HA-antibody (Invitrogen). The membrane with the blotted proteins was than stained with amido black to reveal equal loading of GST and GST fusion proteins.

### Co-immunoprecipitation (CoIP)

Transfected 293T cells were lysed in 200 µl lysis buffer (50 mM HEPES pH 7.4, 150 mM NaCl, 10 mM MgCl2, 5 mM EDTA, 10% glycerol, 1% Triton X-100, 5 mM sodium orthovanadate, 5 mM glycerol 2-phosphate and a mixture of protease inhibitors) per well. PC12 cells were lysed with RIPA buffer. Aliquots were either directly analyzed by western-blotting or were used for protein binding studies. For use in co-immunoprecipitation, lysates were incubated with anti-FLAG antibody conjugated to agarose beads (Sigma) at 4°C for 3 hours to overnight. The beads were extensively washed with lysis buffer and analyzed by western-blotting with monoclonal and polyclonal anti-FLAG antibodies (Sigma) and anti-HA antibody (Invitrogen).

### Direct binding assay

Transfected or untransfected 293T cells were lysed with RIPA buffer and subjected to immunoprecipitation with anti-FLAG antibody conjugated to agarose beads (Sigma) at 4°C overnight. Precipitates were washed thoroughly with RIPA buffer and with RIPA buffer supplemented with 0.1–0.5% sodium dodecyl sulfate or 500 mM NaCl. Precipitates were resuspended in lysis buffer (50 mM HEPES pH 7.4, 150 mM NaCl, 10 mM MgCl2, 5 mM EDTA, 10% glycerol, 1% Triton X-100, 5 mM sodium orthovanadate, 5 mM glycerol 2-phosphate and a mixture of protease inhibitors) and mixed with 250 ng Pin1 protein that had been expressed and purified from *E.coli*. Samples were incubated at 4°C overnight. After sedimentation, precipitates were washed with RIPA buffer and analyzed by western-blotting with anti-Pin1 antibody together with input samples. Subsequently, the membrane was stained with amido black to demonstrate equal loading.

### Immunofluorescence and confocal microscopy

PC12 cells grown on sterilised poly-D-lysine-coated glass coverslips were fixed with 3.7% formaldehyde for 15 minutes at 37°C. Fixed cells were washed twice with PBS and permeabilised with 0.2% Triton X-100 (BioRad) in PBS for 15 minutes at room temperature. Blocking was carried out with 2% bovine serum albumin and 7% fetal bovine serum in PBS for 30 to 60 minutes at room temperature. Cells were incubated with anti-BNIP-H (Buschdorf et al. 2006) and anti-Pin1 (Santa Cruz Biotechnology) antibodies in blocking solution. Samples were washed three times with 0.1% Triton X-100-containing PBS before incubation with Alexa Fluor 488-conjugated donkey anti-mouse IgG and Alexa Fluor 594-conjugated goat anti-rabbit IgG (both from Molecular Probes). P19 cells were processed the same way except that cells were post-fixed with 3.7% formaldehyde at 4°C overnight. Pictures were taken with a confocal microscope (Zeiss).
